# IDO/kynurenine pathway in cancer: possible therapeutic approaches

**DOI:** 10.1186/s12967-022-03554-w

**Published:** 2022-08-02

**Authors:** Eslam E. Abd El-Fattah

**Affiliations:** Department of Biochemistry, Faculty of Pharmacy, Delta University for Science and Technology, Gamasa, Egypt

**Keywords:** Indoleamine 2,3-dioxygenase, Kynurenine, Lung cancer, IDO/kynurenine inhibitors, IDO/kynurenine stimulator

## Abstract

Cancer is one of the leading causes of death in both men and women worldwide. One of the main changes associated with cancer progression, metastasis, recurrence, and chemoresistance is the change in the tumor immune microenvironment, especially immunosuppression. Cancer immunosuppression appears in multiple forms, such as inhibition of immuno-stimulant cells with downregulation of immuno-stimulant mediators or through stimulation of immuno-suppressive cells with upregulation of immunosuppressive mediators. One of the most immunosuppressive mediators that approved potency in lung cancer progression is indoleamine 2,3-dioxygenase (IDO) and its metabolite kynurenine (Kyn). The current review tries to elucidate the role of IDO/Kyn on cancer proliferation, apoptosis, angiogenesis, oxidative stress, and cancer stemness. Besides, our review investigates the new therapeutic modalities that target IDO/Kyn pathway and thus as drug candidates for targeting lung cancer and drugs that potentiate IDO/Kyn pathway and thus can be cancer-promoting agents.

## Introduction

Indoleamine 2,3-dioxygenase (IDO) is a 403-amino-acid cytosolic heme-containing enzyme that degrades tryptophan (Trp), an essential amino acid, through the kynurenine (Kyn) pathway (KP). IDO causes Trp to be degraded, which is required for adequate Kyn concentrations and other important cellular activities. Endothelial cells in the placenta and lung, epithelial cells in the female vaginal canal, and mature dendritic cells (DC) in lymphoid organs express IDO in normal human tissues. Endometrial and cervical carcinomas have the most considerable IDO-expressing cells among human malignancies, followed by kidney, lung, and colon cancers. IDO activity has been linked to acquired immunological tolerance, including the suppression of T-cell activation and the activation of regulatory T cells (Tregs), which can allow tumor cells to avoid immune surveillance [1, 2].

The plasma Kyn to Trp ([Kyn]/[Trp]) ratio is frequently used to express or reflect the activity of the extrahepatic IDO [3].

Tang et al. [4] found that as P53 is inactivated in the majority of cancer types, which accounts for the rise in IDO level, p53 may partially dampen IDO signaling in lung cancer cell migration. Additionally, dinaciclib was discovered to be an indirect KP inhibitor and was proven to cause IDO inhibition [5].

### Physiological and pathological conditions that favor the anabolic/catabolic pathways of l-tryptophan

Physiologically, Trp is an amino acid required for the production of proteins and cellular survival. Trp can be found in several protein-rich meals, including eggs, cheese, and meat. Trp serves as a coenzyme in the important metabolic pathways nicotinamide adenine dinucleotide (NAD) and nicotinamide adenine dinucleotide phosphate (NADP) and is the precursor of the synthesis of Kyn and serotonin. L-tryptophan supplements have been used, and Trp has been researched as a potential treatment for a range of neuropsychiatric disorders by altering the generation of melatonin [6].

Pathologically, Trp deficiency results in depression, behavioral changes, cognitive problems, and mood disturbances [6]. On the other hand, Eosinophilia-myalgia syndrome has been linked to Trp poisoning (EMS). Intense, incapacitating myalgias and severe peripheral eosinophilia are characteristics of EMS [7]. Interferon-gamma (IFN-γ), the classic antitumor-associated T cell effector cytokine, and the agonists of the toll-like receptors 9 (TLR9) and 4 (TLR4), CpG DNA and lipopolysaccharide (LPS), respectively, are among the strongest agonists of IDO transcription [8].

L-tryptophan is converted to serotonin, melatonin, protein, and Kyn through various anabolic/catabolic activities. The rate-limiting breakdown of the Trp indole ring 2,3-double bond and incorporation of molecular oxygen is catalyzed by IDO1 and tryptophan 2,3-dioxygenase (TDO). The ultimate result of this reaction is *N*-formylkynurenine, which is converted to l-Kyn quickly and spontaneously. Downstream intermediates of the latter catabolite include 3-HK, 3-hydroxyanthranilate (3-HAA), and quinolinic acid, all of which alter immunological responses [9].

Even though IDO1 and TDO catalyze Trp, their quaternary structures, expression in normal vs. altered tissue, and regulation are very different. While monomeric IDO1 can cleave both d- and l-Trp, homotetrameric TDO is enantiomer-specific and can only catabolize l-Trp. IDO1 was the only IDO known to function at the 2,3 double bond until 2007. The novel paralog, IDO2, was then discovered by three different groups. While the IDO1 and IDO2 genes are 43 percent homologous and located next to each other on chromosome 8, the Km of human IDO1 and IDO2 for l-Trp is 20.90 3.95M and 6809 917M, respectively, showing that the latter enzyme has a significant drop in activity. This is particularly intriguing because both gene products include the residues necessary for Trp catalytic activity. Also noteworthy is that mouse IDO2 has higher enzymatic activity than the human homolog, although genetic depletion of mouse IDO2 does not affect systemic Kyn levels, in stark contrast to IDO1-deficiency [10, 11].

It was once considered that IDO1 served as an innate immune effector to limit the quantity of Trp required for microbial development since it was elevated in response to infection. Munn and Mellor changed their minds after demonstrating that in vivo injection of the IDO1 inhibitor 1-methyl tryptophan (1-MT) caused T cell-dependent fetal allograft rejection. According to a recent study, IDO1-expressing macrophages, DC, and tumor cells limit T cell growth. Downstream stress-response pathways such as general control non-depressible 2 (GCN2) and mammalian target of rapamycin (mTOR), both essential regulators of amino acid sufficiency, mediate IDO1 responses. The GCN2 kinase phosphorylates the alpha subunit of translation initiation factor 2 alpha, resulting in translation inhibition when amino acid deficiency induces an increase in total uncharged tRNA levels. The ability of GCN2-activated plasmacytoid DC to limit T cell proliferation in vivo via an IDO1-dependent mechanism was first observed [12].

In a mouse papilloma model, it was later shown that genetic deletion of IDO1, but not GCN2, inhibited skin carcinogenesis, implying that additional critical pathways existed downstream of IDO1 activation. IDO1-mediated Trp depletion reduced mTOR, a crucial immunoregulatory kinase that could be reactivated in vitro by treatment with 1-MT, a Trp mimic [13].

### Tumor risk factors and IDO expression

Pertovaara et al. [14] found that Smoking subjects have lower IDO enzyme activity, which suggests that the known immunostimulatory effects of smoking may be caused by a decrease in IDO-dependent immunosuppression. Jiang et al. [15] found that in all of the examined brain areas of ethanol addiction/withdrawal animals, IDO1 was discovered to rise at both the mRNA and protein levels. In behavioral tests, alcohol-exposed mice had gradually impaired memory function along with anxious and sad behavior. In the hippocampus, cerebral cortex, and amygdala of ethanol addiction/withdrawal mice, however, it was discovered that KYN was expressed more, 5-hydroxytryptamine (5-HT) was expressed less, and 3-hydroxykynurenine (3-HK) and kynurenic acid (KA) were expressed abnormally.

### Regulation of IDO1

Numerous redundant mechanisms lead to IDO1 expression and activity in the literature. IDO1 expression is induced by pro-inflammatory signals such as IFN-γ, CpG DNA, and LPS. T tumor necrosis factor-alpha (TNF-α), IL-6, and IL-1β are only a few cytokines that work together to boost IDO1 expression. Prostaglandin E2, the oncogene c-KIT, and the tumor suppressor Bin1 are all IDO1 modulators. Wnt5 also controls IDO1 activity in DC via β-catenin signaling while maintaining continuous expression in several cancer cell lines via an AhR-IL-6-STAT3 (Signal Transducer And Activator Of Transcription 3) signaling loop, according to new research [16, 17].

In gastrointestinal cancers, lung cancer, glioma, melanoma, prostate cancer, and pancreatic cancer, over-activation of the Kyn pathway, particularly IDO, predicts poor prognosis [18].

### Factors that regulate IDO-2 and TDO gene expression and enzymatic activity

Regarding IDO-2, IDO2 mediates the autoreactive B cell response driving arthritis through an IDO1-independent mechanism [19]. IDO2 suppression by 1-MT raises the possibility that the IDO2 enzyme plays a role in tumors' ability to evade the immune system [20]. IDO2 mRNA expression might be induced in human mesenchymal stem cells and certain cancer cells by IFN-γ. At the same time, it was discovered that LPS, prostaglandin E2, and interleukin-10 (IL-10) all contributed to the activation of IDO2. It's interesting to note that the expression of IDO2 could be induced by the aryl hydrocarbon receptor (AHR), indicating that the promoter of the ido2 gene contains an AHR responsive region [21].

Regarding TDO, The anti-TDO-2 antibody was nevertheless able to recognize the protein generated by the mutant with the 9 bp deletion (tdo-2 (PLD)), even though it was likely lacking exactly three amino acid residues. Higher Trp levels and lower Kyn levels were observed in samples from animals whose TDO-2 proteins had large truncations as well as in samples from the PLD mutants, indicating that the three amino acids missing in this mutant are necessary for TDO-2 enzymatic activity. The deletions resulted in complete knockout mutations [22].

### IDO, Kyn, and Trp levels in cancer

Onesti et al. [23] observed that notably increased plasmatic Kyn, Trp, and their ratio in breast cancer patients compared to healthy controls. Onesti et al. [23] observed In contrast to tumors with hormone receptors, patients with hormone receptor-negative disease have lower plasmatic Trp and a greater Kyn/Trp ratio. Compared to other histologies, lobular tumors had the lowest ratio. Lower Trp levels and higher Kyn/Trp ratios were linked to more advanced tumors, respectively. Higher Kyn readings were related to pathological complete response. Trp, Kyn, and Kyn / Trp ratios in plasma did not predict survival. Suzuki et al. [24] found that lung cancer patients had higher IDO activity, and higher IDO activity was linked to more advanced stages of the disease.

This meta-analysis comprised a total of 31 papers. In general, there was a strong correlation between high IDO expression and poor OS (Overall survival) [25]. IHC staining revealed that 63.2 percent of bladder cancer tissues had high levels of IDO1 expression, compared to 29.4 percent of the adjacent normal tissues. This difference was statistically significant between the bladder tumor tissues and the adjacent normal tissues, and IDO1 expression was significantly correlated with tumor size, T stage, and N stage. [26].

IDO expression was found in all but three patient tumor samples, in all but four autologous non-malignant lung tissues, in three of the nine human lung cancer cell lines, and 28 patients with diverse primary lung cancers. The relative expression of IDO was considerably lower in lung cancer cell lines (4.7 ± 11.1) compared to all patient tumor samples (p = 0.006) and autologous non-affected lung tissues (p = 0.027) [27].

The plasma Kyn/Trp ratio is frequently used to express or reflect the activity of the extrahepatic Trp-degrading enzyme IDO was added as a method of assessment of the IDO/Kyn pathway.

### IDO angiogenesis

**Fig. 1:**
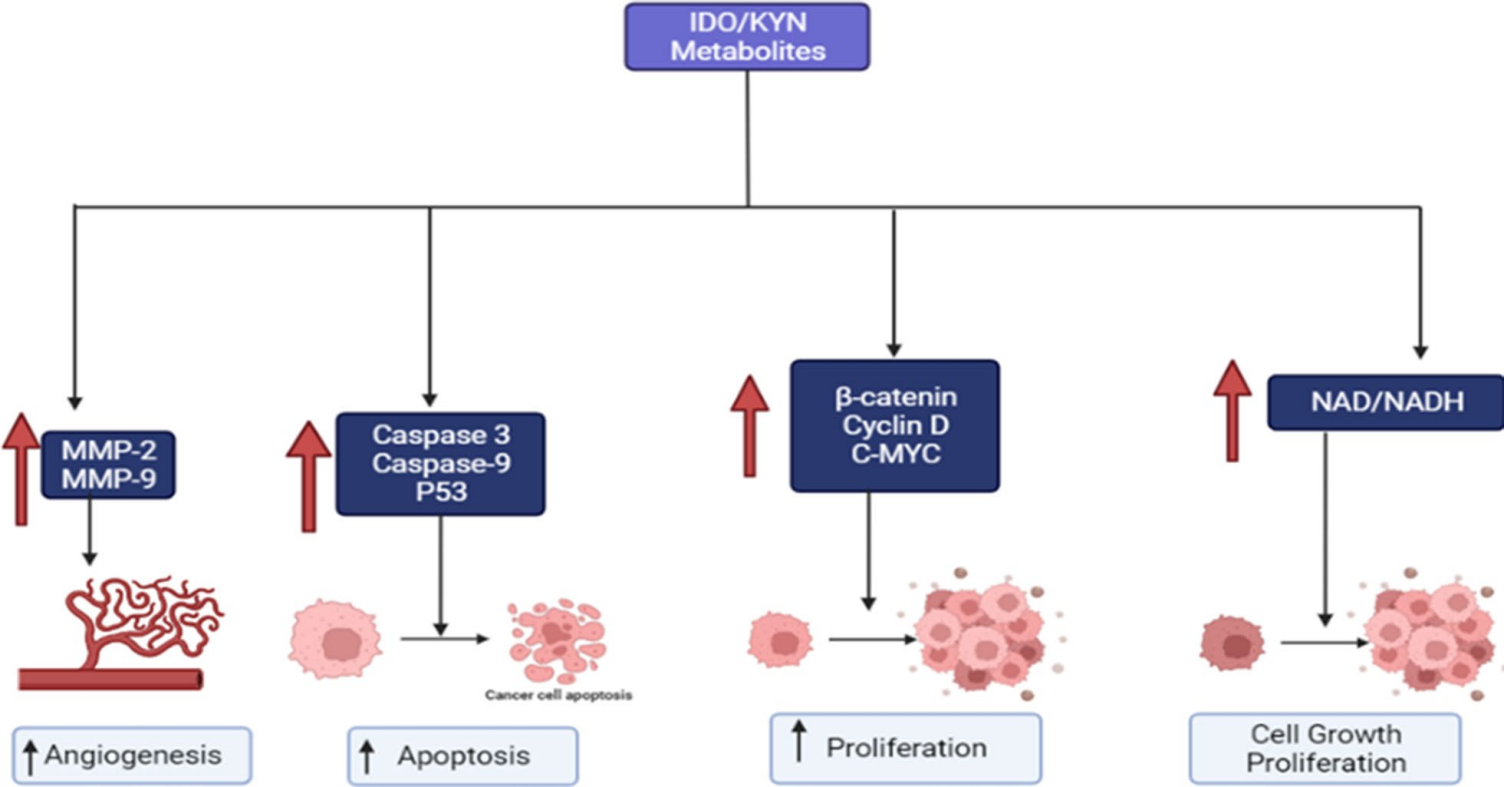
The effect of the IDO/Kyn pathway on cancer proliferation, apoptosis, and angiogenesis. MMP-2: Matrix metalloproteinase, NAD: Nicotinamide adenine dinucleotide

IDO has no influence on lewis lung cancer cell proliferation, but it can boost adhesion and promote invasion, metastasis, and vasculogenic mimicking abilities. It can also help vascular endothelial cells become more angiogenic, implying that IDO's immunological role is not the only one. In lewis lung cancer cells, overexpression of IDO enhanced Janus tyrosine kinase 2 (JAK-2) and STAT3 phosphorylation and up-regulated the production of Matrix metalloproteinase-2 (MMP-2) and MMP-9, two essential genes involved in invasion and metastasis [28] (Fig. 1). The microvascular density (MVD)-CD105 level was higher in IDO positive tissue than in IDO negative tissue (10.90 vs 7.46) [29]. CD34 and CD146 protein expression were dramatically reduced in experimental tumor tissues from IDO1 short hairpin RNA (shRNA) treated mice [30]. Gao et al. [31] found that in colon cancer patients, among the TDLN without metastases, a higher density of IDO + cells was documented in 21/60 cases (35%).

The numbers of invasion cells transfected with IDO1 Small interfering RNA (siRNA), GL2 siRNA, and control cells, respectively, were 108.6676.658/well, 341.33316.773/well, and 333.33316.442/well, indicating a significant difference [30]. Pagano et al. [32] observed that in a mouse colon cancer xenograft, activation of GPR35 (G Protein-Coupled Receptor 35), a target protein for KYNA and 3-HAA, is linked to increased neoangiogenesis, tumor tissue remodeling, and tumor development. IDO1/TDO expression was found to be positively linked with aquaporin expression, suggesting that IDO1/TDO may play a role in glioma cell motility [33]. Comparing these bladder cancer cells to the control group, IDO1 knockdown decreased their capacity to migrate, and si-IDO1 transfection dramatically decreased the expression of N-cadherin and vimentin proteins as compared to the si-NC group [26].

### IDO and apoptosis

The Kyn triggers apoptosis in cells with considerably higher Caspase-3 and Caspase-9 activity [34]. Zhong et al. [35] mentioned that TUNEL research revealed that IDO1–/– fibrosis mice had lower rates of apoptotic cell death in liver tissues than WT fibrosis mice, demonstrating that IDO causes apoptosis. Transgenic expression of IDO increased renal tubular epithelial cells (TEC) death without pro-inflammatory cytokine exposure, suggesting that IDO is involved in TEC damage [36]. The JAK-STAT1 pathway is required for IDO activation by IFN-gamma, and its induction triggers 3OHKyn-mediated apoptosis in HLE-B3 cells [37]. IDO suppression lowered the expression of p53 and p21 in T-cells in mixed lymphocyte reactions (MLRs), indicating that IDO has a pro-apoptotic effect [38]. Sas et al. [39] mentioned that IDO inhibition lowers NAD+ production, which leads to cell death (Fig. 1).

### IDO and proliferation

The Ki67 index (mitotic index) and overall survival were favorably linked with IDO1/TDO expression. In human colon cancer cells, diminished IDO1 activity reduced nuclear and activated β-catenin, transcription of its target genes (cyclin D1 and Axin2), and, ultimately, proliferation [33, 40].

In human colon cancer cells, inhibiting IDO1 activity reduced nuclear and activated β-catenin, transcription of its target genes such as cyclin D1, and, ultimately, proliferation [41]. IDO1 and other Kyn pathway genes were decreased by dinaciclib, a cyclin-dependent kinase (CDK) inhibitor [5]. In a colorectal model, Kyn treatment caused fast and dose-dependent Protein kinase B (Akt) activation, as demonstrated by elevations in pAKT S472 and phosphorylated PRAS40 pT246, a direct target of Akt activity [42]. IDO reduced the expression of ζ-chain, c-Myc, LDH-A, and GLS2 in MLRs when compared to untreated MLRs [38]. Compared to usual settings, KYNA serum deprivation decreased the growth of U-343 MG cells [43] (Fig. 1).

The CCK-8 assay revealed that IDO1 knockdown dramatically reduced the proliferation of T24 and UMUC3 cells, and after 2 weeks of culture, the colony formation rate of cells with IDO1 knockdown was significantly reduced in comparison to the control group [26]**.**

### IDO and oxidative stress

3-HK produces hydrogen peroxide and other reactive oxygen species (ROS), linked to neuronal cell death in a brain region implicated in the pathogenesis of neurodegenerative diseases such as Alzheimer's disease [44]. IDO-1 is induced in response to oxidative stress as well as inflammation. The inflammatory mediators up-regulate IDO-1 expression; TNF-α, IL-1β, IL-2, and IL-6 [45].

Accordingly, IDO1 activation and resultant L-Trp metabolism along the Kyn pathway can protect against oxidative stress via promoting de novo NAD+ synthesis in a human astroglioma cell line exposed to hydrogen peroxide (H2O2) [46]. 3-OH-KYN is transferred into cells by neutral amino acid transporters. Only after interaction with cellular xanthine oxidase is 3-OH-KYN capable of producing sufficient amounts of ROS, such as superoxide radicals, hydrogen peroxide, and hydroxyl radicals, inducing internucleosomal DNA cleavage leading to apoptosis [47].

### IDO and hypoxia

Indoleamine 2, 3-dioxygenase is an enzyme that metabolizes Trp which up-regulates degradation of l-tryptophan and increases hypoxia-inducible factor-1 expression [48]. Endogenous Trp derivatives, such as Kyn and ITE (2-(10H-indole-30- carbonyl)-thiazole-4-carboxylic acid methyl ester), may play opposite roles in cancer progression and stemness, regulating OCT4 expression through AhR modulation: accumulation of the low-affinity AhR agonist Kyn in the tumor microenvironment favor carcinogenesis, whereas the high-affinity AhR agonist ITE promotes its binding to the OCT4 promoter to suppress its transcription and, consequently, inducing cell differentiation in U87 glioblastoma neurospheres [49].

### IDO and CSCs

Indoleamine 2, 3-dioxygenase mRNA was enhanced four to sevenfold in spheres compared to adherent counterparts for different cell types. Trp in the media with spherical cells decreased by 3 mg/ml in 24 h, whereas adherent cells declined only 1–1.5 mg/ml [50]. Low et al. [51] found that IDO1 protein expression was upregulated in cervical tumorspheres from HeLa and SiHa cervical cancer cells compared to 2D cultured cells. In addition to the protein level, the IDO1 activity, which was determined by the conversion of Kyn from Trp using Ehlrich reagent, was also increased in HeLa and SiHa tumorspheres in comparison to 2D cultured cells. Compared to control shRNA transduced cells, the colony number of IDO1 knockdown cervical tumorsphere cells derived from HeLa and SiHa cells reduced dramatically as radiation increased [51].

### IDO and cancer energetics (NAD)

Using human MDMs has provided evidence that indicates that an immune-mediated increase in IDO activity does increase NAD biosynthesis concomitantly with an increase in NAD catabolism [52] (Fig. 1).

### IDO/Kyn and cancer immune escape

The main theory linking IDO, TDO, and IDO2 to immunosuppression focuses on how each of them contributes individually and/or collectively to Trp metabolism. This dogma's foundation is the Trp Starvation Theory, which holds that Trp depletion at or below 1M, which is considered to be nearly absolute, promotes the accumulation of uncharged tRNAs, which in turn activates the GCN2 kinase pathway and causes T cells to malfunction. Studies conducted in vitro lend credence to the idea that Trp depletion suppresses the main metabolic regulators mTOR and protein kinase C (PKC) in cancer cells, hence promoting autophagy and Treg formation, respectively.

Through the production of Kyn and other downstream derived metabolites, Trp degradation may also inhibit immune cell activity. Kyn activates the AhR, a ligand-activated transcription factor that has significant effects on immune cells and is implicated in the differentiation of inducible Tregs, in vitro and further requires co-treatment with transforming growth factor-beta (TGF-β). Kyn metabolites from the downstream pathway, such as KA, xanthurenic acid (XA), and cinnabarinic acid (CA), interact with AhR and may influence the immunological response. Contrastingly, it has been shown that Trp catabolites can also cause CD4+ T cell death. Kyn, 3-HK, and 3-HAA inhibit T cell growth concurrently with apoptotic induction. This data was independently proving that Kyns selectively trigger the apoptosis of murine thymocytes and Th1-cells, but not Th2-cells, in vitro. Maintaining peripheral lymphocyte homeostasis and preventing the buildup of autoreactive and/or inflammatory lymphocytes may depend on Kyns' immunoregulatory effects on several lymphocyte subsets [8].

### Drugs that inhibit IDO/KYN pathways

#### 1-methyl-dl-tryptophan (1-MT)/(indoximod)

Indoximod is similarly effective in suppressing IDO1 enzymatic activity in human monocyte-derived DCs [53, 54].

### Epacadostat

Epacadostat is an IDO1 selective inhibitor with little activity against IDO2 that is currently in clinical development and is expected to be the first IDO1 inhibitor to achieve registration approval. In its early phase I/II trials, epacadostat demonstrated preliminary promising anticancer effects when used in conjunction with anti-programmed cell death protein (anti-PD-1) drugs such as pembrolizumab and nivolumab in individuals with advanced malignant melanoma [55, 56].

### Doxorubicin (DOX) and navoximod combination

Doxorubicin treatment of breast tumor cells resulted in IDO1 upregulation. These findings showed other potential methods of DOX impact on tumor repression, particularly in metastatic breast cancer, where traditional DOX treatment causes a variety of adverse effects and consequent therapeutic failure [57]. Recent improvements in understanding the immunological changes caused by chemotherapy and advances in combining checkpoint IDO1 inhibitors with conventional chemotherapy are promising for increasing numbers of cancer [58]. Navoximod is a potent IDO pathway inhibitor with promising pharmacological effects for treating cancer-related immunosuppression [59].

### Linrodostat

Linrodostat is a small molecule effectively and specifically inhibits IDO1, preventing Trp from being converted into the immunosuppressive Kyn [60] to lower serum Kyn levels and inhibit tumor, IDO1's heme is particularly labile, and linrodostat inhibits IDO1 by binding to the heme-free (apo) form of the enzyme [60, 61].

### Imatinib

Imatinib stimulated CD8+ T cells and triggered Treg death within the tumor via decreasing IDO1 expression on tumor cells. In a mouse model of the spontaneous gastrointestinal stromal tumor (GISTs), IDO1 regulation was shown to contribute significantly to imatinib's anti-cancer effects [62].

### Nimesulide

Epithelial malignancies could over-express cyclooxygenase-2 (COX-2) like non-small lung cancer (NSLC). Consequently, it gives it malignancy and metastatic characters [63]. COX-2 enhances immunosurveillance escape which is demonstrated by the finding that inhibiting COX-2/PGE2 in animals with lung cancer reduces Treg-cell frequencies while increasing the frequency of anti-tumor effector T cells [64]. A cancer study in an animal model derived us from understanding the relationship between COX-2 and IDO1. In the tumor site, the inhibition of COX2 can down-regulate IDO1 expression and fall serum Kyn levels [65, 66]. Nimesulide, a selective COX-2 inhibitor, lowered IDO1 mRNA/protein and decreased Kyn production, implying that total IDO1 inhibition was caused by both reduced IDO1 gene transcription and hindered IDO1 catalytic activity [67].

### Metformin

Patients with insulin resistance were accompanied by elevated Kyn metabolites before hyperglycemia signs appeared [68]. It was found that patients on the metformin regimen had normal Trp metabolism and Kyn metabolites [69].

### Dinaciclib

Dinaciclib is a CDK inhibitor that could suppress the Kyn pathway in glioblastoma multiforme (GBM) as well as head and neck squamous cell carcinomas (HNSCC) [5].

### Selective serotonin reuptake inhibitor treatment with probiotic bacteria *Lactobacillus plantarum*

Selective serotonin reuptake inhibitor (SSRI) and probiotic bacteria *Lactobacillus plantarum 299v* could significantly decrease Kyn level, associated with cognitive functions improvement [70].

### Galanal

Methanol extraction of Myoga flower buds contains galanal which was found to significantly inhibits IDO1 activity [71].

### The galanthamine–memantine combination

The galanthamine–memantine combination could affect the receptors of alpha7 nicotinic acetylcholine and *N*-methyl-d-aspartate; moreover, it can inhibit KA in the Kyn pathway [72].

### M4112

Although M4112 could block IDO1 activity in vitro and there was a safety dose margin, the Kyn plasma level did not change and may need further investigations [73].

### Candesartan

The candesartan derivatives were associated with IDO 1 inhibition through the enzymatic active site and not through the haem region [74].

### Desipramine

Desipramine inhibits the expression of IDO1 and IDO2 in peripheral blood mononuclear cells (PBMCs) [75].

### Simvastatin and sildenafil

It is possible to conclude that co-administration of simvastatin and sildenafil had provided a neuroprotective effect against irradiation-induced brain injury. The protection mechanism was through NO donor/tetrahydrobiopterin (BH4). Besides, the combination offered anti-inflammatory and anti-oxidant properties with IDO/KYN modulation [76].

### Lacosamide

Anti-epileptic lacosamide was used to treat partial-onset seizures in children (> 1 year), and adults had significant against a neuroinflammation-mediated model of concurrent seizures with depression by Kyn levels reduction in hippocampal [77].

### Eicosapentaenoic acid

Eicosapentaenoic acid is one of the omega-3 fatty acids extracted from animals and marine plants. It had a significant effect on Kyn levels, decreasing its level with increasing T-cells survival. The anti-tumor action of eicosapentaenoic acid contributed to IDO1 expression blockage [78].

### Sulfonamide

Sulfonamides have a variety of pharmacological properties in vivo, including anti-carbonic anhydrase and anti-t dihydropteroate synthetase, which allows them to be used to treat a variety of diseases such as diuresis, hypoglycemia, thyroiditis, inflammation, bacterial infection, and glaucoma. Sulfonamide has significant potent IDO1 inhibitory action with similar efficacy to Epacadostat in *vivo* Lewis lung cancer [79].

### Carbidopa

Carbidopa had a similar structure to phenylhydrazine, which is an IDO1 inhibitor. Carbidopa could significantly decrease in vitro and in vivo pancreatic cancer cell proliferation [80].

### PCC0208009

PCC0208009 had significant selective pain suppressing, as it acts as a potent, selective IDO1 inhibitor, effective in treating neuropathic pain [81].

### Lavender oil

The mechanism of action of lavender oil and its derivatives, linalool, α-pinene, and limonene involved in the catabolism of IDO1 and neopterin production through GTP, cyclohydrolase-I, and IFN-γ [82].

### Ketoprofen and sertraline combination

Ketoprofen is a nonsteroidal anti-inflammatory drug (NSAID) while sertraline is SSRI antidepressant drug. Sertraline and ketoprofen had significant results in decreasing IDO1 levels and inflammation and immunity modulation in major depression disorder. The ketoprofen provided a synergetic effect to sertraline towards IDO1 inhibition and benefited action for T-helper and T-reg cells [83]**.**

### Chloroquine

The 4-aminoquinoline-based medicines chloroquines are primarily used to treat malaria. In in vitro studies of human PBMCs, chloroquine had interfered with IFN-γ. Moreover, it could stimulate neopterin synthesis and Trp catabolism. For that reason, chloroquine had significant anti-inflammatory properties to be used clinically [84].

### 2-hydrazinobenzothiazole

In vitro, a potent inhibitor for IDO1 was 2-hydrazinobenzothiazole, while phenylhydrazine bound to haem and inhibited IDO1 [85].

### Nitroglycerin

Nitroglycerin is a vasodilator that is commonly used to treat angina chest discomfort. An animal study revealed that nitroglycerin could significantly down-regulate the Kyn level [86].

### Nitric oxide (NO)

Nitric oxide produces cGMP, which induces vascular relaxation. NO could successfully block IDO1 [87].

### Curcumin

Based on IFN-γ stimulation of IDO1 expression, curcumin could inhibit IDO1 and suppress immunological t-cells. Consequently, the downregulation of IDO1 in DC is a vital mechanism of immunological changes induced by curcumin which could be used in cancer therapy [88].

### Flavonoids

Flavonoids bind non-competitively with IDO1 confirmed by plasmon resonance assays. It was significantly used in cancer immunotherapy [89].

### Progesterone

Progesterone could significantly inhibit the IFN-γ Kyn pathway induction, decreasing excitotoxin quinolinic acid concentration. It could promote neuroprotection and reduce neopterin. It provided an interpretation of gender variations in the inflammatory response [90].

### Nicotine

Smokers had significantly lower activity of IDO1 than non-smokers with unknown mechanisms of smokers' immunostimulatory action [91].

### *N*-acetyl-cysteine

*N*-acetylcysteine (NAC) is the mainstay of therapy for acetaminophen toxicity. The significant cellular protection from Kyn causes programmed cell death with anti-oxidant NAC. It also inhibits NK cells mediated ROS pathway in addition to the IDO1 blocking effect [92].

### Lithium

Lithium has been the therapy of choice for bipolar disorder (BD) for more than six decades. Lithium could inhibit IDO1 action in primary cells in immortalized human microglial cells. Moreover, it increased the production of IL-10. Lithium blocks the Kyn inflammatory pathway in the microglia part of the human brain [93].

### Melatonin

Melatonin is produced by the pineal gland during the night in reaction to darkness. Exogenous melatonin could inhibit neuroinflammation through attenuating IDO expression (94).

### Hydroxyamidines

In vitro analysis, Hydroxyamidines have significantly inhibited the metabolism of Trp in colon carcinoma and pancreatic carcinoma cells and in vivo cancers in lymph node drainage. INCB024360 has a significant IDO1 inhibitor with desirable clinical outcomes in cancer patients [95].

### Benserazide

Benserazide is a dihydroxyphenylalanine (DOPA) decarboxylase inhibitor that does not penetrate CNS and is used as an addition to levodopa in treating Parkinsonism. Benserazide inhibits the Kyn metabolism peripherally and treats olanzapine-induced metabolic syndrome [96].

### Caffeine

Caffeine is the most used stimulant in the world. There was a significant correlation between anxiety caused by caffeine and the Kyn level. The Kyn level is proportional to anxiety peaks [97].

The following table (Table 1) summarizes the above-mentioned drugs that inhibit IDO/Kyn pathway.

**Table 1:** Drugs that inhibit IDO/Kyn pathway

	Drug	Main target	Species	Model or cells	References
1	Indoximod	IDO1 (enzyme activity)	Human	Breast cancer Melanoma	[53, 54]
2	Epacadostat	IDO1 (enzyme activity)	Human	Melanoma	[55, 56]
3	Linrodostat	IDO1 (enzyme activity)	Human	Advanced cancers	[60, 61]
4	Imatinib	IDO1 (gene expression)	Murine	Gastrointestinal stromal tumor	[62]
5	Nimesulide	COX-2/IDO1 (gene expression)	Human (In vitro)	AML	[67]
6	Metformin	Kyn (signal transduction)	Human	Insulin resistant case	[69]
7	Dinaciclib	CDK (gene expression)	Human (In vitro)	Glioblastoma multiforme (GBM) Head and neck squamous cell carcinomas (HNSCC)	[5]
8	SSRI + Lactobacillus Plantarum	Kyn (enzyme–substrate binding)	Human	Major depression	[70]
9	Galanal	IDO1 (enzyme activity)	Human (In vitro)	Acute leukemia	[71]
10	M4112	IDO1 (enzyme activity)	Human	Solid tumors	[73]
11	Candesartan	IDO1 (enzyme activity)	Human	Hypertension	[74]
12	Desipramine	IDO1 (gene expression)	Human Murine	PBMCs	[75]
13	Simvastatin and Sildenafil	IDO1 (enzyme activity)	Murine	Irradiation-induced brain injury	[76]
14	Lacosamide	Kyn (enzyme activity)	Murine	Depression	[77]
15	Eicosapentaenoic acid	IDO1 (gene expression)	Murine (In vitro)	Breast cancer Melanoma	[78]
16	Sulfonamide	IDO1 (enzyme activity)	Murine (In vitro)	Lewis lung cancer	[79]
17	Carbidopa	IDO1 (enzyme activity)	Human and Murine (In vitro and in vivo)	Pancreatic cancer Liver cancer	[80]
18	PCC0208009	IDO1 (enzyme activity)	Murine	Neuropathic pain	[81]
19	Lavender oil	IDO1 (enzyme activity)	Human (In vitro)	PBMC	[82]
20	Ketoprofen and sertraline combination	IDO1 (gene expression)	Human	Depression	[83]
21	Chloroquine	IDO1 (enzyme activity)	Human In vitro	Leukemia	[84]
22	2-hydrazinobenzothiazole	IDO1 (enzyme activity)	Murine (In vitro)	Lung cancer	[85]
23	Nitroglycerin	IDO1 (gene expression)	Murine	Migraine	[86]
24	Nitric oxide (NO)	IDO1 (enzyme activity)	Human murine	Mononuclear phagocytes	[87]
25	Curcumin	IDO1 (gene expression)	Murine	DCs	[88]
26	Flavonoids	IDO1 (enzyme activity)	Human	Cervical cancer cells	[89]
27	Progesterone	Kyn (enzyme activity)	Human	Monocyte-derived macrophages	[90]
28	Nicotine	IDO1 (enzyme activity)	Human	Peripheral blood	[91]
29	*N*-acetyl-l-cysteine	Kyn (enzyme activity)	Human	NK cells	[92]
30	Lithium	IDO1 (enzyme activity)	Human	Brain cortical biopsy	[93]
31	Melatonin	IDO1 (gene expression)	Murine	Neuroinflammation	[94]
32	Hydroxyamidines	IDO1 (enzyme activity)	Murine (In vitro)	Colon carcinoma	[95]
33	Benserazide	IDO1 (enzyme activity)	Murine	Weight Gain, Insulin Resistance, and Dyslipidemia	[96]
34	Caffeine	Kyn (enzyme activity)	Human	Anxiety	[97]

### Drugs that stimulate IDO/KYN pathways

#### Statin

Asthmatic patients were administered inhaled corticosteroids; when they were given statins as statins enhance the anti-inflammatory effect, IDO1 activity was altered through increasing IDO induction [98].

#### Nandrolone decanoate

**Fig. 2:**
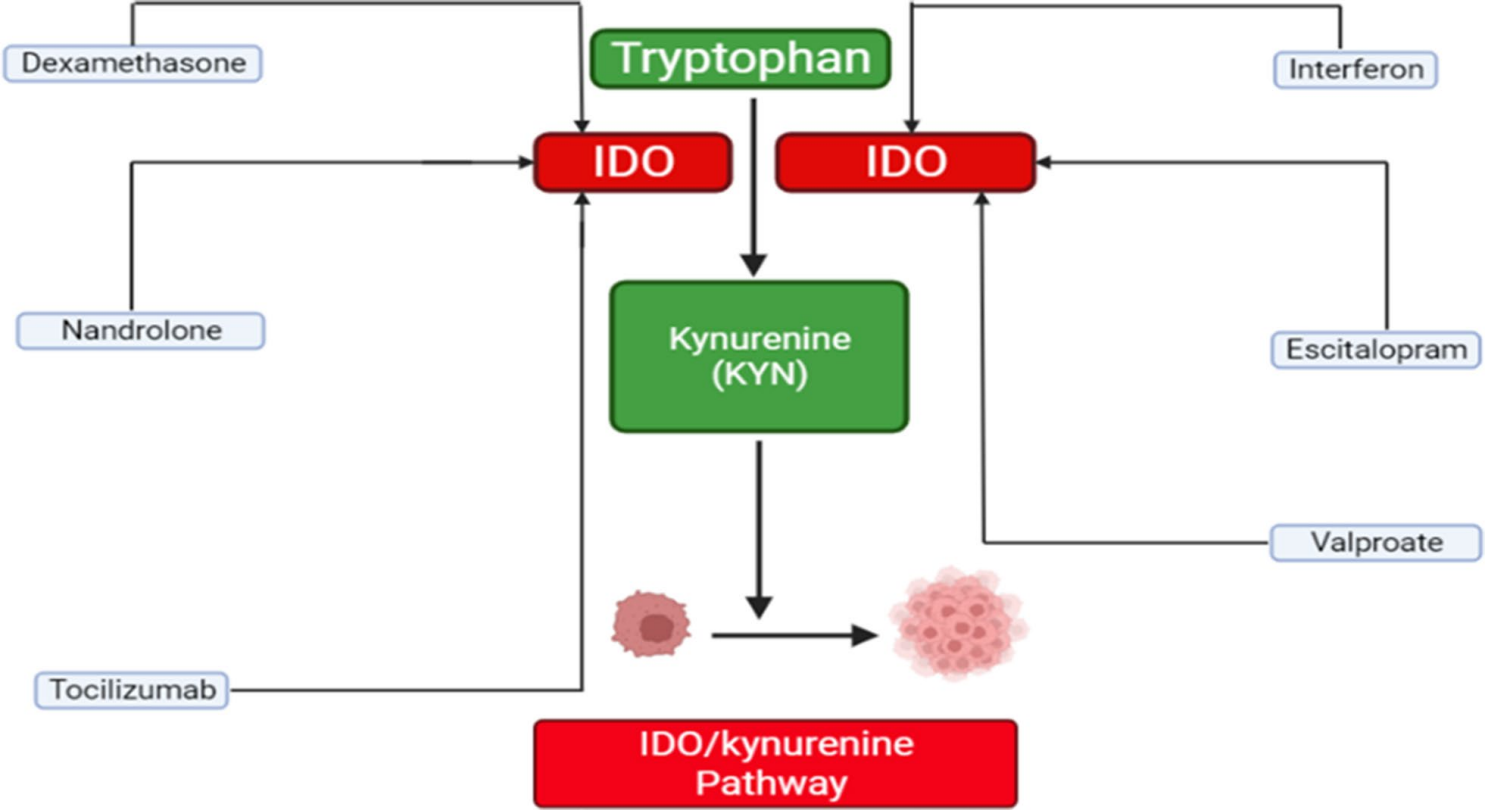
Drugs that stimulate the effect of the IDO/Kyn pathway. IDO: indoleamine 2,3-dioxygenase; Kyn: kynurenine (Kyn)

Nandrolone decanoate is one of the most often abused anabolic androgenic steroid molecules globally. However, it comes with a slew of side effects. Nandrolone decanoate could promote IDO1 activity, increasing Kyn concentration in the brain [99] (Fig. 2).

#### Escitalopram

Antidepressant selective serotonin reuptake inhibitor, Escitalopram, is a drug that is used to treat major depressive disorder and anxiety disorders. The synthesis of neurotoxic Kyn metabolites could be significantly performed by escitalopram; moreover, it could inhibit inflammatory response [100] (Fig. 2).

#### Dexamethasone

Dexamethasone belongs to the corticosteroid family that is used to treat chronic obstructive pulmonary disease, severe allergies, rheumatic disorders, and asthma. Dexamethasone could increase the effect of IFN-γ as a super-stimulation of IDO1. It could modulate the immune system and regulate the metabolism of Trp [101] (Fig. 2).

#### Valproate

Valproate sodium is an anti-epileptic and mood stabilizer that could stimulate increasing Kyn in the brain with its similar mechanism of action. Valproate could displace Trp from albumin, consequently increasing the Kyn of the brain [102] (Fig. 2).

#### Interferon

The only type II interferon, IFN-γ, is innate and adaptive immune responses. In human peripheral blood monocytes, IFN-γ had significant stimulation of IDO and more induction of Trp catabolism. Besides, IFN-γ had a higher effect than interferon-alpha [101] (Fig. 2).

#### Tocilizumab

Tocilizumab is an anti-human IL-6 receptor (IL-6R) monoclonal antibody authorized for rheumatoid arthritis. It blocks IL-6 signaling by binding soluble IL-6R and membrane IL-6R. Tocilizumab could provide Trp-derived catabolites and block IL6 activities [103] (Fig. 2).

Table 2 summarizes the above-mentioned drugs that stimulate IDO/Kyn pathway.

**Table 2:** Drugs that stimulate IDO/Kyn pathway

	Drug	Main target	Species	Model or cells	References
1	Statin	IDO1 (Gene expression)	Human	Asthma	[98]
2	Nandrolone decanoate	IDO1 (IDO activity)	Murine	Depression	[99]
3	Escitalopram	Kyn (enzyme–substrate binding)	Human	Neurotoxicity	[100]
4	Dexamethasone	IDO1 (IDO activity)	Human	Peripheral blood monocytes	[101]
5	Valproate	Kyn (enzyme–substrate binding)	Murine	Brain	[102]
6	Interferon	IDO1 (IDO activity)	Human	Peripheral blood monocytes	[101]
7	Tocilizumab	IDO1 (IDO activity)	Human	Diabetes	[103]

Besides these are some of the clinical trials that target the IDO/Kyn pathway as mentioned in https://www.clinicaltrials.gov (Table 3).

**Table 3:** Clinical trials that target IDO/Kyn pathway

	NCT Number	Title	Conditions
1	NCT03047928	Combination Therapy With Nivolumab and PD L1/IDO Peptide Vaccine to Patients With Metastatic Melanoma	Metastatic Melanoma
2	NCT01219348	IDO Peptide Vaccination for Stage III-IV Non-Small-cell Lung Cancer Patients	NSCLC Lung Cancer
3	NCT02967419	The Study of the Relationship Between TWEAK/Fn14, JAK/STAT3, and IDO in the Immune Microenvironment of Endometrium in Repeated Implantation Failure	Repeated Implantation Failure
4	NCT01397916	IDO Activity in Patients With Chronic Lymphocytic Leukemia (CLL)	CLL

## Conclusion

As IDO and its metabolite Kyn are potential targets for cancer control due to their immunosuppressive effect, IDO/Kyn also induces its carcinogenic effect on proliferation, apoptosis, angiogenesis, metastasis, oxidative stress, and cancer stemness potentiality. Besides, many drugs inhibit IDO/KYN pathway in different diseases, which can be tested for their effectiveness against cancer progression. On the other hand, different drugs induce the IDO/KYN pathway, which may potentiate cancer progression and thus look for alternatives in case of cancer risk with other comorbidities.

## Data Availability

Not applicable.
